# Distinct amyloid distribution patterns in amyloid positive subcortical vascular cognitive impairment

**DOI:** 10.1038/s41598-018-34032-3

**Published:** 2018-11-01

**Authors:** Hyemin Jang, Jong-Yun Park, Young Kyoung Jang, Hee Jin Kim, Jin San Lee, Duk L. Na, Young Noh, Samuel N. Lockhart, Joon-Kyung Seong, Sang Won Seo

**Affiliations:** 1 Departments of Neurology, Samsung Medical Center, Sungkyunkwan University School of Medicine, Seoul, Korea; 2 Neuroscience Center, Samsung Medical Center, Seoul, Korea; 3 School of Biomedical Engineering, Korea University, Seoul, Republic of Korea; 4 Department of Neurology, Kyung Hee University Medical Center, Seoul, Korea; 5 Stem Cell & Regenerative Medicine Institute, Samsung Medical Center, Seoul, Korea; 6 Department of Neurology, Gachon University Gil Medical Center, Incheon, Korea; 7 Department of Health Sciences and Technology, GAIHST, Gachon University, Incheon, Korea; 8 Department of Internal Medicine, Wake Forest School of Medicine, Winston-Salem, NC USA; 9 Department of Health Sciences and Technology, SAIHST, Sungkyunkwan University, Seoul, Korea; 10 Department of Clinical Research Design & Evaluation, SAIHST, Sungkyunkwan University, Seoul, Korea

## Abstract

Amyloid-β (Aβ) and cerebral small vessel disease (CSVD) commonly coexist. They can occur independently by chance, or may interact with each other. We aimed to determine whether the distribution of Aβ in subcortical vascular cognitive impairments (SVCI) patients can be classified by the underlying pathobiologies. A total of 45 ^**11**^C-Pittsburgh compound B PET positive (PiB(+)) SVCI patients were included in this study. They were classified using a new cluster analysis method which adopted the Louvain method, which finds optimal decomposition of the participants based on similarity of relative Aβ deposition pattern. We measured atherosclerotic cerebral small vessel disease (CSVD) markers and cerebral amyloid angiopathy (CAA) markers. Forty-five PiB(+) SVCI patients were classified into two groups: 17 patients with the characteristic Alzheimer’s disease like Aβ uptake with sparing of occipital region (OccSp) and 28 patients with occipital predominant Aβ uptake (OccP). Compared to OccSp group, OccP group had more postive association of atherosclerotic CSVD score (*p* for interaction = 0.044), but not CAA score with occipital/global ratio of PiB uptake. Our findings suggested that Aβ positive SVCI patients might consist of heterogeneous groups with combined CSVD and Aβ resulting from various pathobiologies. Furthermore, atherosclerotic CSVD might explain increased occipital Aβ uptakes.

## Introduction

Amyloid-β (Aβ) and cerebral small vessel disease (CSVD) pathology are two of the most common contributors to late-life cognitive impairment. Previous studies have suggested that Aβ and CSVD are associated with one another^1–6^. In fact, about 30% of subcortical vascular cognitive impairment (SVCI) patients have significant brain Aβ deposition, a hallmark of Alzheimer’s disease (AD)^7^. Aβ and CSVD have common risk factors such as old age, hypertension and diabetes mellitus. Aβ and CSVD pathology can occur independently by chance, or they may interact each other. For example, cerebral amyloid angiopathy (CAA) may possibly contribute to altered vascular reactivity, eventually accelerating CSVD. Alternatively, atherosclerotic CSVD may decrease clearance of amyloid via perivascular lymphatic drainage^6^. These possible pathobiologies might contribute to heterogeneous patterns of Aβ accumulation, depending on which is more predominant in patients with cognitive impairment.

Aβ is usually widely distributed in the association neocortex by the onset of cognitive symptoms^8^. Typical late-onset AD patients frequently show increased Aβ uptake in frontal, temporal and parietal cortical regions with relative sparing of occipital regions^9^. Given that the two predominant vascular lesions in AD are CAA and arteriosclerosis/lipohyalinosis^4^, the effect of these vascular pathologies on Aβ accumulation has been of a great interest. CAA patients exhibit increased Aβ uptake in occipital regions where CAA is known to predominantly occur^10–12^. Atherosclerotic CSVD might also induce Aβ deposition in posterior regions, given a previous study showing that SVCI patients had relatively higher Aβ deposition in posterior area than AD patients^13^. Another study from our group demonstrated that white matter hyperintensities were associated with Aβ uptake in the posterior region only in APOE4 non-carriers^14^. This study raised two possible hypotheses for this association; (1) posterior circulation may be vulnerable to vascular injury leading to more Aβ deposition, or (2) it might be related to the topography of CAA.

In this study, we tried to classify [^11^C] Pittsburgh compound B (PiB) positive (+) SVCI patients, characterized by extensive CSVD and significant Aβ burden measured by PiB positron emission tomography (PET) according to PiB uptake pattern by employing a novel clustering method. We also investigated whether different subtypes show distinct associations of CAA and atherosclerotic CSVD markers with PiB uptake. We hypothesized that PiB uptake patterns in PiB(+) SVCI might be classified into several subgroups depending on their possible pathobiology. That is, given that Aβ may independently develop regardless of ischemia, Aβ might accumulate in an AD-like characteristic pattern yielding an occipital sparing (OccSp) PiB(+) SVCI; conversely, when Aβ and CSVD happen to interact with each other, Aβ might be more preferentially deposited in occipital regions, producing an occipital predominant (OccP) PiB(+) SVCI. We further hypothesized that CAA and atherosclerotic CSVD markers would be more strongly associated with increased occipital PiB uptake in OccP PiB(+) SVCI compared with OccSp PiB(+) SVCI patients.

## Results

### Subject Demographics

The final study sample consisted of 45 patients with PiB(+) SVCI. The mean age of all participants was 77.3 ± 5.3 years old, and the frequency of APOE4 carriers was 46.7%.

### Cluster Analyses of PiB PET

**Figure 1:**
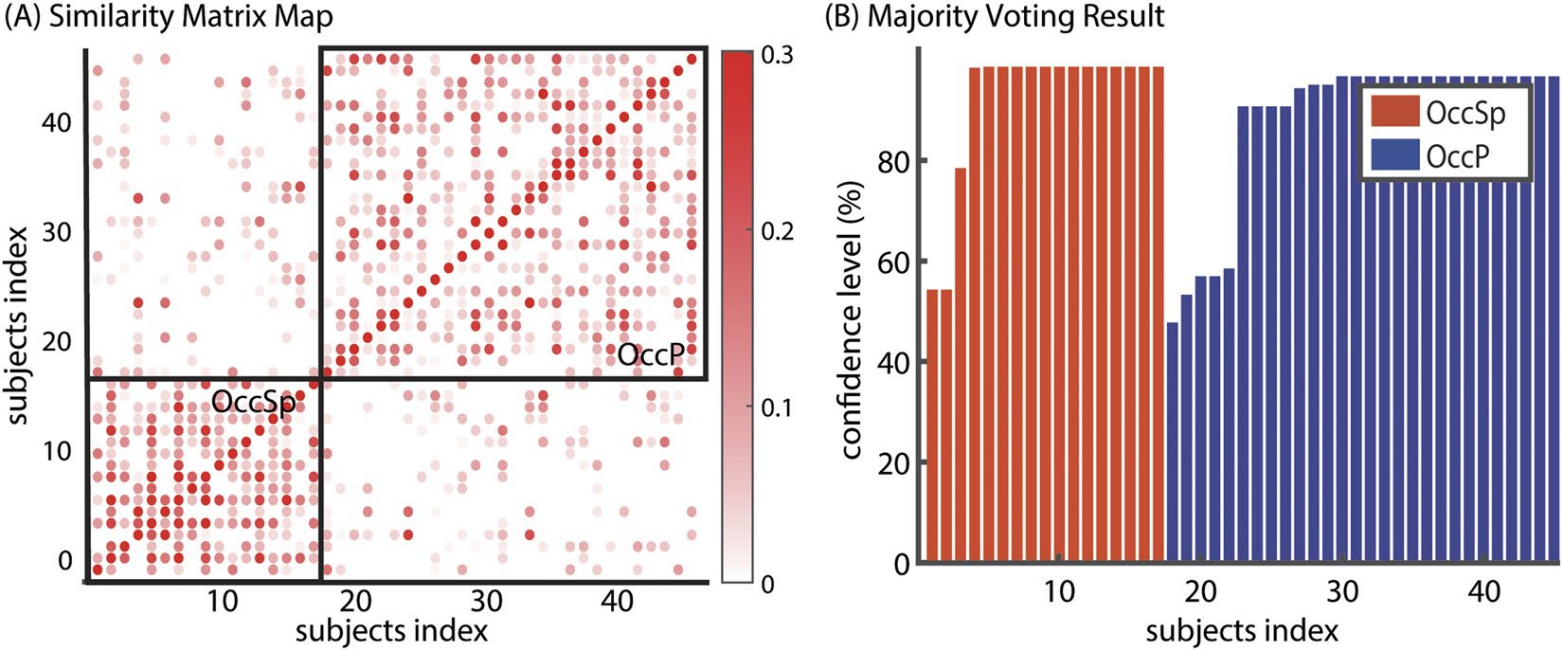
Similarity matrix map and majority voting result of extracted modular organization. (**A**) Similarity matrix shows high intra-modular correlation, with sparse inter-modular correlation. We computed correlation coefficient between all pairs of SVCI subjects. We reordered subjects by same clustered subtypes and drew borderlines. (**B)** Majority voting result shows high reproducibility (93.14%) across 1000 repetitions. Abbreviations: OccSp = Occipital sparing; OccP = Occipital predominant.

The whole participants were classified into two distinct clusters with highly correlated relative Aβ deposition pattern within cluster, while distinct between clusters (Fig. 1(A)). The clustering results showed not only a remarkable modularity value (0.6186) but also a high confidence level (93.14%) (Fig. 1(B)).

**Figure 2:**
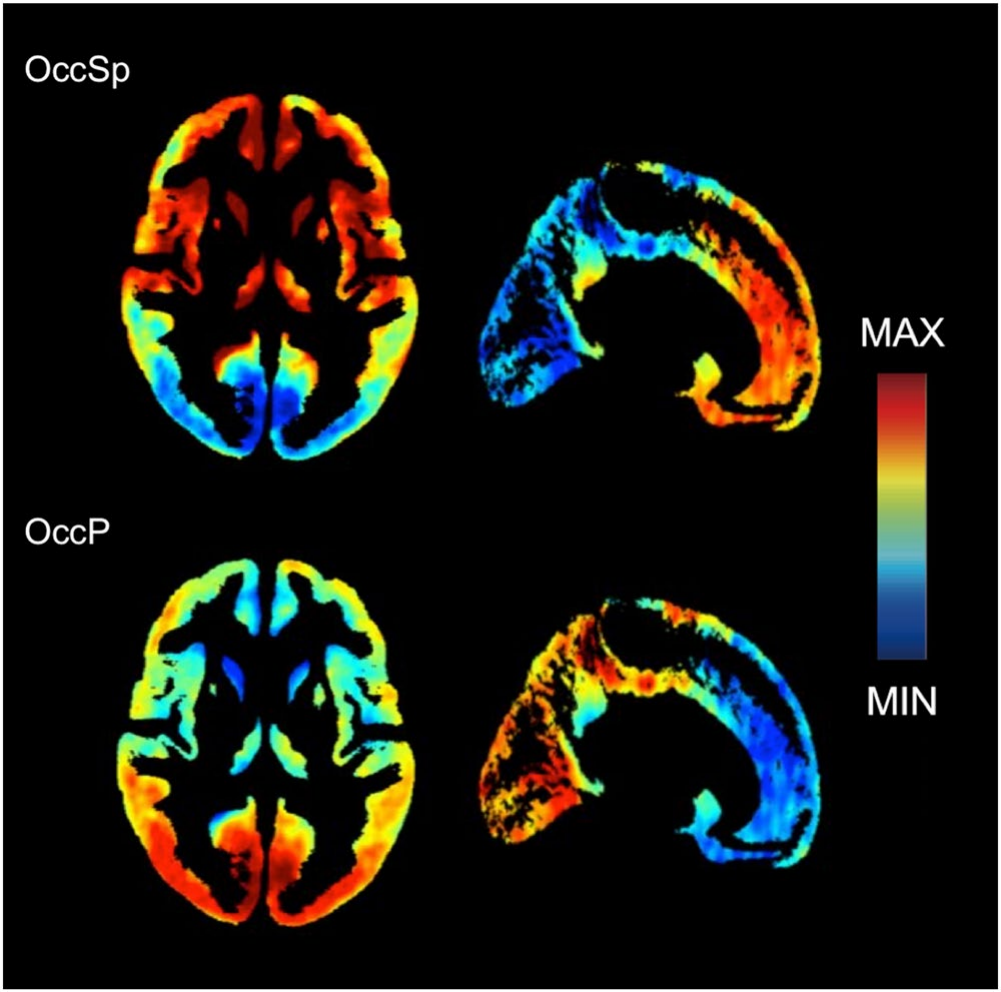
Two distinct distribution patterns of PiB uptake in PiB(+) SVCI. Deposition maps show the mean of the normalized (z-score) grey matter SUVR in each subtype. The OccSp groups had higher uptake in anterior regions and the OccP had higher uptake in posterior regions. Abbreviations: OccSp = Occipital sparing; OccP = Occipital predominant; PiB = Pittsburgh compound B; SUVR, Standardized uptake value ratio; SVCI = subcortical vascular cognitive impairment.

Figure 2 shows two distinct distribution patterns of PiB uptake in PiB(+) SVCI. Seventeen participants exhibited higher PiB uptake in the frontal, anterior and inferior temporal and medial and lateral parietal regions with some sparing occipital region (OccSp), while the remaining 28 participants had higher PiB uptake prominently in the posterior temporal and occipital regions (OccP). As shown in Supplementary Figure S1, the OccSp group showed higher frontal/global SUVR ratio than OccP group (1.05 ± 0.04 versus 0.98 ± 0.04, p < 0.001), while the OccP group showed greater occipital/global SUVR ratio than OccSp group (0.94 ± 0.05 versus 1.05 ± 0.08, p < 0.001).

### Comparisons of APOE Genotyping and Clinical Features

There were no differences in age, education, gender and a prevalence of vascular risk factors between OccSp and OccP groups. The OccSp group was more likely to have *APOE*4 than the OccP group (70.6% versus 32.1%, p = 0.012) (Table 1). Although there were only four patients who fulfilled the modified Boston criteria for probable CAA, all of them belonged to OccP group. There were no significant differences in neuropsychological test results in any cognitive domains between the two groups (Table 1). In addition, there were no significant differences in atherosclerotic CSVD and CAA burden between the two groups as well (Table 2).

**Table 1:** Demographic and clinical characteristics of participants.

	OccSp SVCI with PiB(+) n = 17	OccP SVCI with PiB(+) n = 28	*p*
Age (years)	76.9 ± 5.8	77.5 ± 5.2	0.761
Gender (male, %)	15 (64.7%)	18 (64.3%)	0.977
Education (years)	8.7 ± 5.9	10.7 ± 5.6	0.267
Vascular risk factors			
Hypertension (%)	11 (64.7%)	17 (60.7%)	0.789
Diabetes (%)	5 (29.4%)	5 (17.9%)	0.467
Hyperlipidemia (%)	4 (23.5%)	9 (32.1%)	0.737
Cardiac disease (%)	3 (17.6%)	6 (21.4%)	0.999
Stroke (%)	5 (29.4%)	3 (10.7%)	0.226
APOE genotyping			
ApoE e4 (%)	12 (70.6%)	9 (32.1%)	0.012
ApoE e2 (%)	2 (11.8%)	3 (10.7%)	0.913
Neuropsychological tests^a^			
Attention	8.2 ± 2.0	8.3 ± 1.8	0.574
Language	17.3 ± 5.6	17.1 ± 5.0	0.460
Visuospatial function	23.8 ± 10.1	22.6 ± 10.9	0.321
Memory	37.3 ± 14.6	36.0 ± 25.1	0.874
Frontal/executive function	19.5 ± 8.1	21.4 ± 10.0	0.479

Values are presented as mean ± SD or number (percentage) as appropriate.

Abbreviations: APOE = Apolipoprotein E; n = number; OccSp = Occipital sparing; OccP = Occipital predominant; PiB, Pittsburgh compound B; SVCI = subcortical vascular cognitive impairment;

Clustering analyses classified SVCI patients with PiB positive into two groups by PiB PET distribution; Occipital sparing (OccSp SVCI with PiB(+)) versus Occipital dominant (OccP SVCI with PiB(+)) group.

^a^Age, gender, and education were used as covariates.

**Table 2:** Atherosclerotic CSVD and CAA Score in Clustered Groups.

	OccSp SVCI with PiB(+) n = 17	OccP SVCI with PiB(+) n = 28	*p*
Atherosclerotic CSVD burden			
Deep MBs	0 (0,2)	0 (0,1)	0.383
Lacunes	4 (1,9)	3.5 (2,9)	0.655
WMH volume, mL	36.4 (24.9,53.1)	36.8 (26.2,46.3)	0.867
BG-PVS grade	2 (1,2)	2 (1,3)	0.936
Atherosclerotic CSVD score	2 (1,2)	2 (1,3)	0.375
CAA burden			
Lobar MBs	0 (0,2)	0 (0,3.5)	0.750
Presence of CSS	1 (5.9%)	4 (14.3%)	0.384
CSO-PVS grade	1 (1,2)	2 (1,3)	0.658
CAA score	1 (0,1)	2 (1,2)	0.107

Values are presented as median (interquartile range) or number (percentage) as appropriate.

Abbreviations: BG-PVS, basal ganglia perivascular space; CAA = cerebral amyloid angiopathy; CSO-PVS, centrum semiovale perivascular space; CSS = cortical superficial siderosis; CSVD, cerebral small vessel disease; MBs = microbleeds; n = number; OccSp = Occipital sparing; OccP = Occipital predominant; PiB, Pittsburgh compound B; SVCI = subcortical vascular cognitive impairment; WMH = white matter hyperintensity.

Clustering analyses classified SVCI patients with PiB positive into two groups by PiB PET distribution; Occipital sparing (OccSp SVCI with PiB(+)) versus Occipital dominant (OccP SVCI with PiB(+)) group.

### Interaction Between atherosclerotic CSVD or CAA Scores and Clustered Groups for Occipital/global PiB SUVR Ratio in PiB(+) SVCI

**Figure 3:**
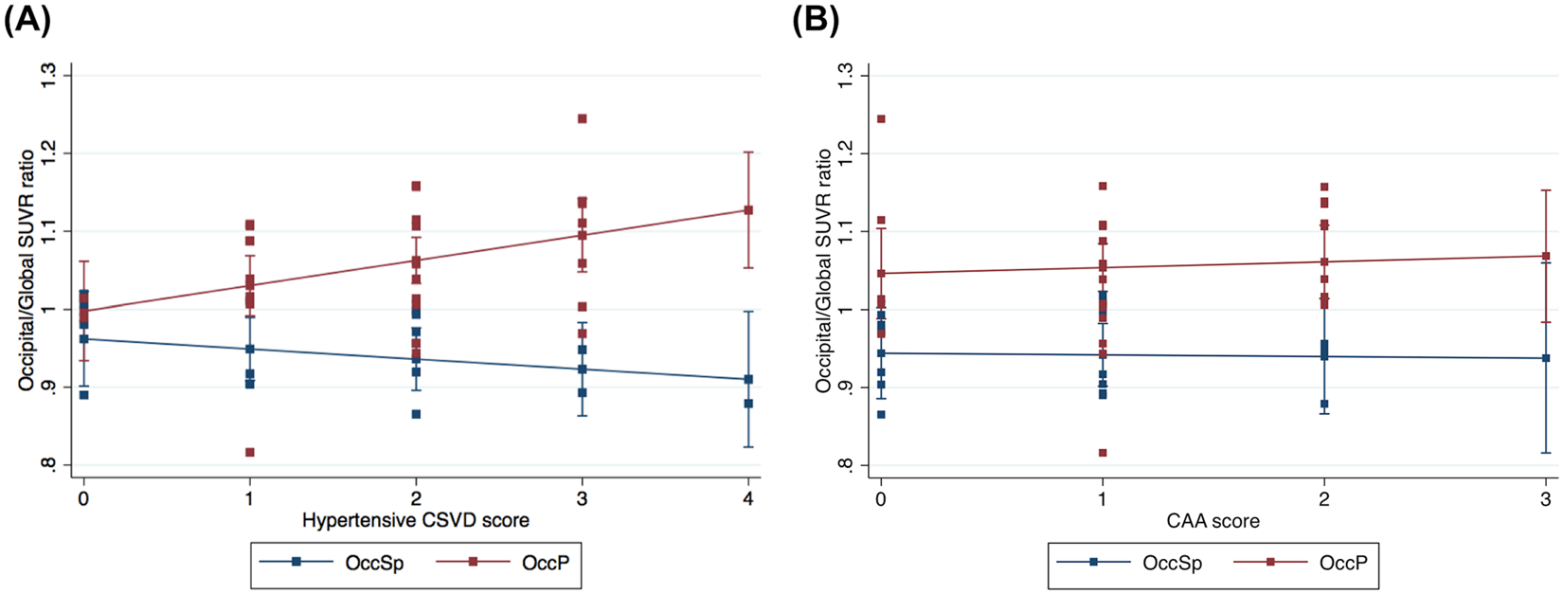
Relationships between occipital/global PiB SUVR and atherosclerotic CSVD (**A**), or CAA scores (**B**) in PiB(+) SVCI patients. Multiple linear regression models were adjusted for age, group (OccP vs. OccSp), and APOE4. There was a significant interaction between atherosclerotic CSVD score and groups (OccP vs. OccSp) for occipital/global PiB SUVR (β (SE) = 0.045 (0.022), *P* = 0.044), while there was no interaction between CAA score and groups (β (SE) = 0.010 (0.034), *P* = 0.777). Abbreviations: CAA = cerebral amyloid angiopathy; CSVD, cerebral small vessel disease; OccSp = Occipital sparing; OccP = Occipital predominant; PiB = Pittsburgh compound B; SUVR = Standardized uptake value ratio; SVCI = subcortical vascular cognitive impairment.

Figure 3 showed the relationships of atherosclerotic CSVD or CAA score with occipital/global PiB SUVR in OccP group and OccSp group. There was an interaction between atherosclerotic CSVD score and clustered groups (OccP group vs. OccSp group) for the occipital/global PiB SUVR ratio (β (SE) = 0.045 (0.022), *P* = 0.044), such that the OccP group had a more postive association between atherosclerotic CSVD score and occipital/global PiB SUVR ratio than the OccSp group (Fig. 3A). However, there was no interaction between CAA score and clustered groups for the occipital/global PiB SUVR ratio (β (SE) = 0.010 (0.034), *P* = 0.777) (Fig. 3B).

## Discussion

In this study, we classified PiB(+) SVCI patients into two groups using a clustering method based on relative Aβ deposition pattern. We found that PiB(+) SVCI patients were clustered into two groups: one with a characteristic AD-like Aβ uptake pattern with sparing of occipital regions (OccSp), and one with occipital predominant Aβ uptake (OccP). We also found that the association between atherosclerotic CSVD score and occipital/global PiB SUVR ratio was more positive in the OccP compared to the OccSp group. Taken together, our findings suggest that the PiB(+) SVCI group consists of heterogeneous groups with distinct pattern of Aβ deposition that might be resulting from various pathobiologies.

We found that patients were categorized more frequently into OccP group (62%) than OccSp (38%) group. The topography of Aβ uptake in the OccSp group was similar to the characteristic patterns of Aβ uptake in AD. OccSp group also showed more frequent *APOE4* allele than OccP group. Considering that the *APOE4* allele is associated with Aβ but not with CSVD^15^, it would be reasonable to expect that Aβ and CSVD in the OccSp group could accumulate independently in the brains of patients. APOE4 is also associated with CAA. However, in the occipital sparing group, the effects of APOE4 on parenchymal Aβ overrides those on vascular Aβ. In contrast, the OccP group exhibited a different pattern of Aβ uptake from AD. Our findings are supported by previous studies showing occipital dominant PiB uptake in PiB(+) SVCI compared to PiB(+) AD patients^13^, and increased occipital PiB uptake related to WMH in PiB(+) *APOE4* non-carrier SVCI patients^14^.

Another noteworthy finding was that there was an interactive effect between atherosclerotic CSVD and clustered groups (OccP versus OccSP) on occipital/global PiB SUVR ratio, such that the association between atherosclerotic CSVD score and occipital/global PiB SUVR ratio was more positive in the OccP group compared to the OccSp group. To our knowledge, the relationship between arteriosclerotic CSVD score and Aβ deposition in the vasculature has not been throughly investigated. However, there are some explanations. First, CSVD might be associated with Aβ deposition in the occipital region because the posterior circulation may be vulnerable to vascular injury and dysfunction of the endothelium leading to BBB disruption (although this study showed hypertensive encephalopathy which is a unique pathological status)^14,16^, which subsequently contributes to decreased Aβ clearance^6,17–22^. The previous study explained that BBB disruption causes leakage of plasma proteins, which may compete with the extracellular fluid containing Aβ for perivascular drainage^6^. This is also supported by the previous experimental study^23^ demonstrating that hypertensive mice models showed a marked Aβ deposition in the same cerebral areas where BBB disruption was detected. Alternatively, it is also possible that occipital dominant PiB uptake pattern might reflect PiB binding influenced by disproportionate perfusion rather than by true Aβ deposition pattern. However, previous studies suggested that CSVD affected hypoperfusion predominantly in the frontal region^24,25^. Further pathological studies should be needed to investigate the pathobiology of occipital dominant Aβ deposition related to CSVD.

We also hypothesized that CAA scores would be more correlated with occipital/global PiB SUVR ratio in the OccP group than the OccSp group based on the previous study^12^. However, we did not find the interaction to be statistically significant. Furthermore, the positive correlation between CAA score and occipital/global PiB SUVR in OccP group was not statistically significant as well. It might be due to small sample size in this study. Alternatively, calculated CAA score might have failed to reflect the true CAA pathology, considering that lobar MBs in the presence of deep MBs are not attributed to underlying CAA pathology. In fact, only four of 45 patients met the modified Boston criteria for probable CAA and all of them were included in the occipital predominant (OccP) group, which suggests that CAA burden might be also associated with posterior Aβ deposition, in line with the previous study^12^. Therefore, this association should be statistically confirmed in the future study including a larger number of CAA patients.

There are several limitations to our study. First, a small sample size might have contributed to low statistical power in analyses. Second, because the categorization of two groups is based on the clustering method and not on an absolute cutoff, the grouping could change depending on the particular study subjects. Further research with more patients from different centers is required to support this study’s finding. Finally, there is a lack of autopsy data, which could have provided pathological confirmation of our findings.

In conclusion, patients were categorized into OccP (62%) and OccSp (38%) group by clustering methods, and the association between atherosclerotic CSVD score and occipital/global PiB SUVR ratio was more positive in the OccP group compared to the OccSp SVCI group. Therefore, our findings suggested that disease patterns in PiB(+) SVCI patients might result from independent or interactive relationships of Aß and CSVD. Finally, these findings are noteworthy for advancing our understanding of mechanisms of combined Aß and CSVD pathologies and for improving treatment decisions for patients with combined Aß and CSVD.

## Materials and Methods

### Participants

We prospectively recruited 45 PiB(+) SVCI patients who visited a Memory Disorder Clinic at Samsung Medical Center from September 2008 to August 2011. SVCI includes both subcortical vascular mild cognitive impairment (svMCI) and subcortical vascular dementia (SVaD). Diagnosis of svMCI was determined using Petersen criteria^26^ with the following modifications^15^: (1) a subjective cognitive complaint by the patient or caregiver; (2) normal Activities of Daily Living (ADL) determined by the instrumental ADL scale as well as clinically; (3) an objective cognitive impairment below the 16^th^ percentile on the Seoul Neuropsychological Screening Battery (SNSB); (4) no dementia; (5) a subcortical vascular feature defined as a focal neurological symptom/sign which includes corticobulbar signs, pyramidal signs, or parkinsonism; and (6) significant ischemia on MRI. Significant ischemia was defined as white matter hyperintensities (WMH) on T2-weighted or FLAIR images that satisfied the following criteria: (1) WMH ≥10 mm in the periventricular white matter (caps or rim) and (2) WMH ≥25 mm (maximum diameter) in the deep white matter, consistent with an extensive white matter lesion or diffusely confluent lesion. SVaD patients were diagnosed by DSM-IV criteria for vascular dementia with significant white matter hyperintensities (WMH), which were defined as above. PiB(+) was established as standardized uptake value ratios (SUVR) ≥ 1.5 (described below).

Patients were assessed by clinical interview and neurological examinations. All patients had laboratory tests including a complete blood count, blood chemistry, vitamin B_12_/folate, syphilis serology, thyroid function tests and apolipoprotein E (*APOE*) genotyping. Structural lesions such as territorial cerebral infarction, brain tumor, hippocampal sclerosis, and vascular malformation were excluded through brain MRI. The Institutional Review Board of Samsung Medical Center approved the study protocol and informed written consent was obtained from each patient. All research was performed in accordance with the relevant guidelines and regulations.

### Neuropsychological Tests

All patients underwent neuropsychological testing using the Seoul Neuropsychological Screening Battery (SNSB)^27,28^. SNSB consists of tests for attention, language, praxis, four elements of Gerstmann syndrome, visuoconstructive function, verbal and visual memory, and frontal/executive function. We derived domain composite scores using the summation of selective tests scores for each subdomain. The raw scores of digit span forward and backward made up the score of the attention domain (total score: 17). The language domain score was derived from the raw score of the Korean version of the Boston Naming Test (total score: 60). The Rey Complex Figure Test (RCFT) copy score was used for the visuospatial function domain (total score: 36). The memory domain was calculated from the sum of immediate-recall score, delayed-recall score, and a recognition score from the Seoul Verbal Leaning and RCFT (total score: 168). Frontal/executive function was assessed using the phonemic and semantic controlled oral word association test (total score: unlimited).

### MRI Acquisition

All participants underwent brain MRI including T2* GRE, three-dimensional (3D) T1 and FLAIR images at Samsung Medical Center using the same kind of 3.0T MRI scanner (Philips 3.0T Achieva; Best, the Netherlands). The following parameters were used for the T2* GRE images: axial slice thickness, 5.0 mm; inter-slice thickness, 2 mm; repetition time (TR), 669 ms; echo time (TE), 16 ms; flip angle, 18°; matrix size, 560 × 560 pixels. We acquired 3D T1 images with the following imaging parameters: sagittal slice thickness, 1.0 mm, over contiguous slices with 50% overlap; TR of 9.9 ms; TE of 4.6 ms; flip angle of 8°; and matrix size of 240 × 240 pixels, reconstructed to 480 × 480 over a field of view of 240 mm. The following parameters were used for the 3D FLAIR images: axial slice thickness of 2 mm; no gap; TR of 11000 ms; TE of 125 ms; flip angle of 90°; and matrix size of 512 × 512 pixels.

### Atherosclerotic CSVD and CAA Scores

White matter hyperintensity (WMH) volume was quantified through fully automated segmentation using FLAIR images, as described in previous studies^29^. A lacune of presumed vascular origin was defined as a lesion ≥3 mm and ≤15 mm in diameter with low signal on T1-weighted images, high signal on T2-weighted images, and perilesional halo on FLAIR images^30^. Microbleeds (MBs) were defined as ≤10 mm in diameter using criteria on 20 axial slices of T2* GRE-MR images according to STRIVE (STandards for ReportIng Vascular changes on nEuroimaging) criteria^30^. Lobar MBs were defined as microbleeds located in lobar areas. Cortical superficial siderosis (CSS) was defined as chronic blood residual products in superficial layers of the cerebral cortex or subarachnoid areas^31^. MRI-visible perivascular spaces in the basal ganglia (BG-PVS) and centrum semiovale (CSO-PVS) were defined and rated using a validated four-point visual rating scale^32,33^ on a single pre-defined slice (first slice above the anterior commissure).

In this study, we used two CSVD scores. We assumed that lacunes, deep MBs^34,35^ and BG-PVS^36^ are more associated with atherosclerosis (age-related and vascular risk factor related CSVD)^37^ and lobar MBs, CSO-PVS, and CSS are more associated with CAA. (Particularly, strictly lobar MBs and CSS are MRI markers for diagnosis of probable CAA according to the modified Boston criteria^38^). Therefore, we calculated atherosclerotic CSVD and CAA scores using these different neuroimaging markers, by modification of previously reported rating score systems^39,40^. We assigned points to atherosclerotic CSVD and CAA scores based on the median value of this group, because PiB(+) SVCI patients showed extensive atherosclerotic CSVD and CAA markers. The atherosclerotic CSVD score (0–4) awards 1 point each for lacunes (if ≥5), deep MBs (if present), BG-PVS (if ≥3) and WMH volume (if ≥36.6 ml), while CAA score (0–3) awards 1 point each for CSS (if present), lobar MBs (if present), and CSO-PVS (if ≥2).

### ^11^C-PiB PET Imaging Acquisition and Imaging Processing

All the patients underwent [^11^C] PiB-PET scanning at either Samsung Medical Center or Asan Medical Center, using a Discovery STe PET/CT scanner (GE Medical Systems, Milwaukee, WI) with identical settings.

Both MR and PET images were co-registered with each other using rigid-body transformation. The T1-weighted MR image of each subject was aligned with the MNI-152 template using a non-linear deformation including translation, rotation, scaling and shearing. After standard space registration, we divided grey matter into 116 regions using the Automated Anatomical Labeling (AAL) atlas^41^. We used a cerebellar reference region^42^ (which did not present group differences) and the cerebral-cortical-region-to-cerebellum-uptake ratio (which is identical to standardized uptake value ratio (SUVR)) to quantify PiB retention. Regional cerebral cortical SUVRs were measured by dividing every cortical region’s SUV by the mean SUV of cerebellar cortex (cerebellum crus1 and crus2). Global PiB retention ratios were assessed from the volume-weighted average SUVR of 28 bilateral cerebral cortical regions. As described above, PiB(+) was defined as global SUVR ≥ 1.5^7,43–45^.

### Imaging Feature-Based Cluster Analysis

We clustered SVCI patients using the clustering method we developed in our previous study^46^. This method finds clusters based on pattern similarity using the Louvain method, which is a state-of-the-art modular extraction method in network science. First, the z-score of amyloid retention intensity was calculated by considering not only each subject’s amyloid deposition pattern but also inter-subject variability among all patients. The relative amyloid deposition is computed using the following formula:$\,{z}_{i}^{Subjec{t}_{j}}=({x}_{i}^{Subjec{t}_{j}}-{\mu }_{i}^{All})/{\sigma }_{i}^{All}$, where ${z}_{i}^{Subjec{t}_{j}}\,$and ${x}_{i}^{Subject{s}_{j}}$ represent relative amyloid deposition and the raw intensity value in the *i*-th voxel (i = 1, 2, …, 401, 354) of the *j*-th subject (j=1, 2, …, 45), respectively. ${\mu }_{i}^{All}$ and ${\sigma }_{i}^{All}$ are the mean and standard deviation of all subjects at the *i*-th voxel, respectively. Second, we computed a Pearson correlation coefficient for each pair of subjects in order to compare amyloid deposition patterns. Third, the Louvain method finds the best modular organization from the resulting similarity matrix. The modularity value Q is maximized when the modular organization has high intra-module correlation and sparse inter-module correlation. We controlled the resolution parameter γ (gamma), which determines the number of clusters, similar to our previous study. Specifically, in the current study, we set γ as 0.4, generating two modules. Also, because the Louvain method finds clusters based on a stochastic approach, the clustering results can be slightly different upon repetition. We therefore adopted a majority voting scheme which labeled the most frequently assigned cluster following a number of repetitions.

### Statistical Analysis

To compare demographic data, *APOE* genotype, and PiB-PET SUVR ratios between clustered groups, Student *t* tests for continuous variables and χ^2^ tests for dichotomous variables were used. Analysis of covariance was used to compare neuropsychological scores between clustered groups, with age, gender and education years as covariates. Atherosclerotic CSVD and CAA burden between clustered groups were compared using *t* tests or χ^2^ tests, appropriately.

To investigate differences in the relationships of atherosclerotic CSVD or CAA score with occipital/global PiB SUVR ratio according to clustered group, a multiple linear regression analysis was performed with occipital/global PiB SUVR as the dependent variable, and atherosclerotic CSVD or CAA scores, age, presence of *APOE4*, clustered groups and an interaction term (clustered groups * atherosclerotic CSVD or CAA scores) as independent variables. All statistical analyses were performed with STATA/SE version 14.0. Statistical significance was defined as two-tailed *p* < 0.05. Data processing was done by SPM version 5 (SPM5), SPM version 8 (SPM8), and Matlab (Version 2014b, Mathworks, Natick, USA).

## Electronic supplementary material

Supplementary Figure

Figure S1
