# Supplementary material for: Distinct amyloid distribution patterns in amyloid positive subcortical vascular cognitive impairment
Source: Sci Rep. 2018 Nov 1;8:16178. doi: 10.1038/s41598-018-34032-3 (PMC6212495; doi:10.1038/s41598-018-34032-3)
Supplement: Supplementary file 1 — Supplementary Figure [file 41598_2018_34032_MOESM1_ESM.pdf]

Distinct amyloid distribution patterns in amyloid positive subcortical vascular cognitive impairment

Hyemin Jang, MD[1,2]*, Jong-Yun Park, MS[3]*, Young Kyoung Jang, MD[1,2]*, Hee Jin Kim, MD, PhD[1,2], Jin San Lee, MD, PhD[4], Duk L.Na, MD, PhD[1,2,5,9], Young Noh, MD, PhD[6,7], Samuel N. Lockhart, PhD[8], Joon-Kyung Seong, PhD[3 †], Sang Won Seo, MD, PhD[1,2,9,10†]

[1]Departments of Neurology, Samsung Medical Center, Sungkyunkwan University School of Medicine, Seoul, Korea

[2]Neuroscience Center, Samsung Medical Center, Seoul, Korea

[3]School of Biomedical Engineering, Korea University, Seoul, Republic of Korea

[4]Department of Neurology, Kyung Hee University Medical Center, Seoul, Korea

[5]Stem Cell & Regenerative Medicine Institute, Samsung Medical Center, Seoul, Korea

[6]Department of Neurology, Gachon University Gil Medical Center, Incheon, Korea

[7]Department of Health Sciences and Technology, GAIHST, Gachon University, Incheon, Korea

[8] Department of Internal Medicine, Wake Forest School of Medicine, Winston-Salem, NC, USA

[9]Department of Health Sciences and Technology, SAIHST, Sungkyunkwan University, Seoul, Korea

[10]Department of Clinical Research Design & Evaluation, SAIHST, Sungkyunkwan University, Seoul, Korea

*These individuals contributed equally to this article as co-first authors

[†] These individuals contributed equally to this article as co-corresponding authors

SUPPLEMENTARY FIGURES

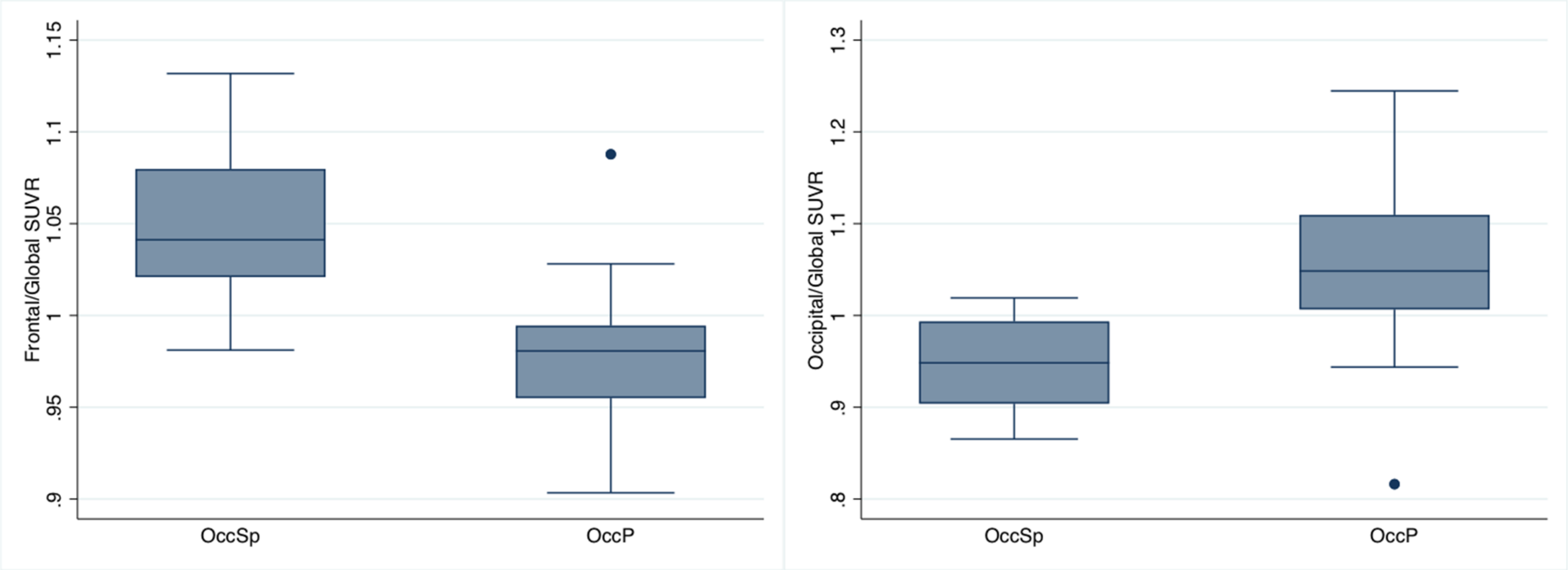

Supplementary Figure S1. Comparison of SUVR ratio of regional amyloid burden

The OccSp SVCI with PiB (+) group showed higher frontal/global SUVR ratio (1.05 ± 0.04 versus 0.98 ± 0.04, p<0.001) while the OccP SVCI with PiB (+) group greater occipital/global (0.94 ± 0.05 versus 1.05 ± 0.08, p<0.001) SUVR ratio.

Abbreviations: OccSp = Occipital sparing; OccP = Occipital predominant; PiB = Pittsburgh compound B; SUVR = Standardized uptake value ratio; SVCI =

subcortical vascular cognitive impairment.
