# Supplementary figures and images for: Distinct amyloid distribution patterns in amyloid positive subcortical vascular cognitive impairment
Source: Sci Rep. 2018 Nov 1;8:16178. doi: 10.1038/s41598-018-34032-3 (PMC6212495; doi:10.1038/s41598-018-34032-3)

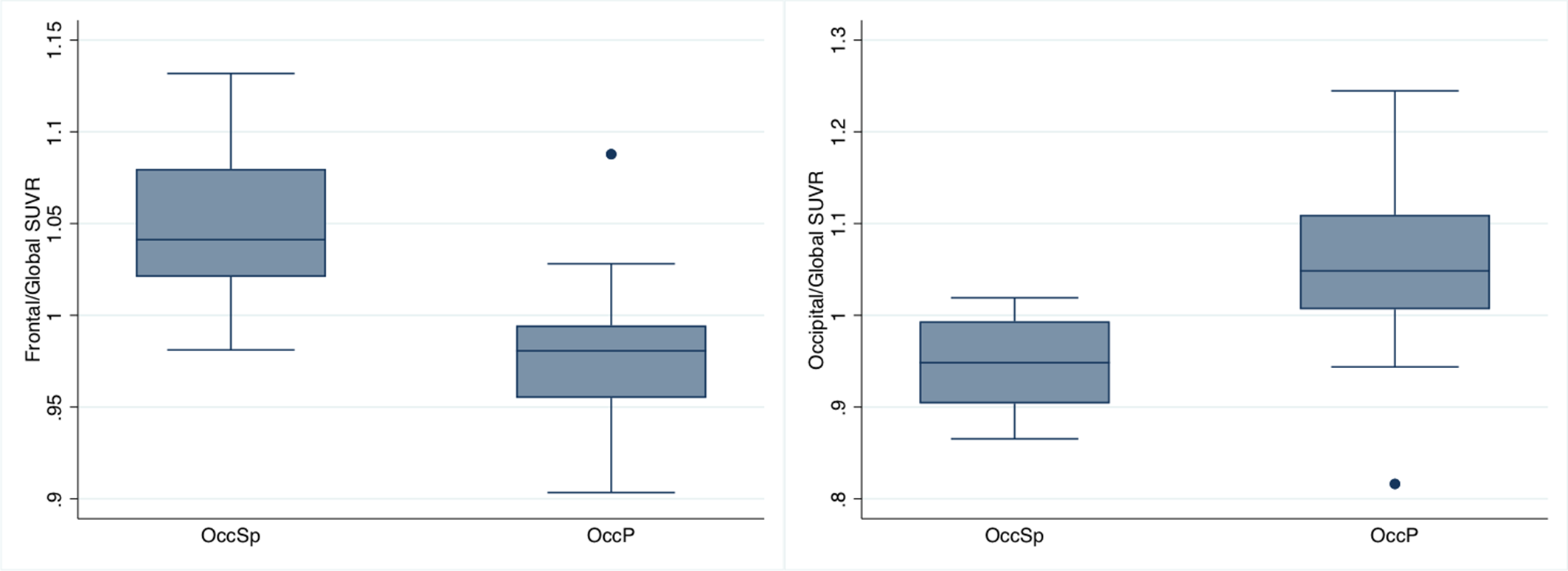

Supplement: Supplementary file 2 — Figure S1 [file 41598_2018_34032_MOESM2_ESM.tif]
